# Women’s access to mental health care in Tunisia

**DOI:** 10.1192/j.eurpsy.2022.2202

**Published:** 2022-09-01

**Authors:** E. Bergaoui, M. Zrelli, N. Staali, M. Moalla, R. Lansari, A. Larnaout, W. Melki

**Affiliations:** Razi Hospital, Psychiatry D, Manouba, Tunisia

**Keywords:** women mental health, Tunisia

## Abstract

**Introduction:**

Tunisia is viewed as an advanced country in terms of women’s rights in the Arab world. However, women are more exposed than men to many specific risk factors which greatly contribute to threaten their mental health.

**Objectives:**

The main objective of this study was to find out the sociodemographic and clinical profiles of women admitted in Razi psychiatric hospital and their access to mental health services.

**Methods:**

A cross sectional and descriptive survey was conducted between March and April 2021 in the department of psychiatry D of Razi Hospital including 40 female inpatients.

**Results:**

The majority of patients had low (37.1%) to moderate (61.9%) socio economic status, with primary education (40%), secondary education (20%) and higher education (28.6%). The majority was unemployed (68.8%). A significant difference was observed between adherence to treatment and family support (p=0.04). It was mainly the father or the husband who was taking care of the patient in 50% of cases. The first psychiatric consultation was 2.68 years after having symptoms. Hospitalization was about 4.94 years later. Twenty five percent of them have seen a tradipractioner before consulting. About 46.87% of patients had conflicts with a member of her family and 15.62% of them were victims of either domestic or family violence. The main diagnoses were mood disorders (31.4%) and schizophrenia (42.9%) Time between symptoms onset and hospitalisation was significantly associated with socioeconomic status (p=0.047) and cultural beliefs (p=0.026).

**Conclusions:**

The protection of women’s mental health is not only a medical challenge but also a cultural and political one.

**Disclosure:**

No significant relationships.

